# Targeting the A3 adenosine receptor to treat hepatocellular carcinoma: anti-cancer and hepatoprotective effects

**DOI:** 10.1007/s11302-023-09925-2

**Published:** 2023-02-13

**Authors:** Pnina Fishman, Salomon M. Stemmer, Avital Bareket-Samish, Michael H. Silverman, William D. Kerns

**Affiliations:** 1 Can-Fite BioPharma Ltd., 10 Bareket St., 49170 Petah Tikva, Israel; 2 Davidoff Cancer Center, Petah Tikva and Sackler Faculty of Medicine, Rabin Medical Center-Beilinson Hospital, Tel Aviv, Israel; 3 BioInsight Ltd., 3056814 Binyamina, Israel

**Keywords:** A3 adenosine receptor, Child–Pugh B, Hepatocellular carcinoma, Liver cancer, Namodenoson, Clinical study

## Abstract

The A3 adenosine receptor (A3AR) is over-expressed in human hepatocellular carcinoma (HCC) cells. Namodenoson, an A3AR agonist, induces de-regulation of the Wnt and NF-kB signaling pathways resulting in apoptosis of HCC cells. In a phase I healthy volunteer study and in a phase I/II study in patients with advanced HCC, namodenoson was safe and well tolerated. Preliminary evidence of antitumor activity was observed in the phase I/II trial in a subset of patients with advanced disease, namely patients with Child–Pugh B (CPB) hepatic dysfunction, whose median overall survival (OS) on namodenoson was 8.1 months. A phase II blinded, randomized, placebo-controlled trial was subsequently conducted in patients with advanced HCC and CPB cirrhosis. The primary endpoint of OS superiority over placebo was not met. However, subgroup analysis of CPB7 patients (34 namodenoson-treated, 22 placebo-treated) showed nonsignificant differences in OS/progression-free survival and a significant difference in 12-month OS (44% vs 18%, *p* = 0.028). Partial response was achieved in 9% of namodenoson-treated patients vs 0% in placebo-treated patients. Based on the positive efficacy signal in HCC CPB7 patients and the favorable safety profile of namodenoson, a phase III study is underway.

## Introduction

Primary liver cancer, and specifically hepatocellular carcinoma (HCC), is a major global health problem due to its incidence, associated mortality, and lack of effective treatment modalities, particularly for patients with moderate or advanced hepatic dysfunction. In the current review, we discuss the preclinical evidence supporting a novel approach for treating HCC, which involves targeting the A3 adenosine receptor (A3AR), and the currently available clinical trial data supporting this approach. We also describe the potential utility of this approach for the treatment of other liver diseases. Lastly, we present the ongoing clinical development program for this approach as well as its current regulatory status.

## Unmet need in advanced HCC with hepatic impairment

Liver cancer is the sixth most common cancer worldwide and the third most frequent cause of cancer-related death globally with approximately 906,000 new cases and 830,000 deaths estimated in 2020 [1]. Liver cancer is more common among males than among females. The cumulative incidence risk (ages 0–74 years) is 1.65% for males and 0.60% for females. The cumulative mortality risks (ages 0–74 years) are 1.49% and 0.55%, respectively [1]. HCC accounts for the majority of primary liver cancers (75–85% of cases) [1]. The main risk factors for HCC include infection with the hepatitis B virus (HBV) or hepatitis C virus (HCV). Other risk factors include heavy alcohol consumption, metabolic liver disease and specifically non-alcoholic fatty liver disease (NAFLD), and exposure to toxins such as aflatoxins and aristolochic acid [2].

The management of HCC takes into consideration not only tumor characteristics, the extent of the disease, and patient comorbidities, but also the patient’s liver function as the benefit of any therapy might be offset by the decline in liver function. For patients diagnosed with early-stage HCC, surgical resection, liver transplantation, and local ablation are potentially curative treatments [2]. Surgical resection is effective; however, more than half of patients experience recurrent disease after such resection [3]. Liver transplantation has the advantage of removing not only the tumor but also the unhealthy liver tissue that is characterized by limited functionality and a tendency to develop HCC within the cirrhotic tissue. Thus, it is an excellent option if the liver dysfunction precludes surgical resection and is a potentially curative approach [2]. Notably, the liver transplantation approach is extremely limited by the restricted number of livers available for transplantation [2]. Percutaneous local ablation (radiofrequency ablation or microwave ablation) works via induction of tumor necrosis by heat delivered into the tumor [2]. Ablation is typically used in patients who are not suitable candidates for surgical resection or liver transplantation due to comorbidities or hepatic dysfunction. It can also be used as a bridge therapy to liver transplantation [4]. For patients diagnosed with intermediate-stage HCC, transarterial chemoembolization (TACE) and transarterial radioembolization (TARE) are effective locoregional treatments. TACE involves intra-arterial infusion of chemotherapy (most commonly doxorubicin, epirubicin, or cisplatin) and occluding the vascular supply to the tumor by delivery of embolization particles into the tumor-feeding artery, leading to ischemic necrosis of the tumor [5]. TARE involves intratumoral brachytherapy that delivers radioactive microspheres into the vascular supply of the tumor, thereby achieving high radiation doses within the tumor [6].

Patients with advanced HCC are typically treated with systemic therapy. The first systemic therapy approved for HCC was the multi-kinase inhibitor sorafenib, which was approved by the United States Food and Drug Administration (FDA) in 2007, as a first-line treatment for unresectable HCC, and since then has been the global standard of care [7]. In 2018, the tyrosine kinase inhibitor lenvatinib was approved by the FDA as an alternative to sorafenib in the first-line setting [8]. Since first-line treatment in advanced HCC often fails due to disease progression or significant toxicity, second and laterline therapies are needed. In recent years, several therapies have been approved for advanced HCC in patients who have been previously treated with sorafenib. These include regorafenib and cabozantinib (multi-target kinase inhibitors), ramucirumab (monoclonal antibody against vascular endothelial growth factor receptor [VEGFR]-2), immunotherapies such as nivolumab and pembrolizumab (monoclonal antibodies against programmed death receptor-1 [PD-1]), and the immunotherapy combination of nivolumab plus ipilimumab (monoclonal antibody against cytotoxic T-lymphocyte antigen 4 [CTLA-4]) (recently reviewed by Rimassa et al. [9]).

Liver dysfunction is often described clinically using the Child–Pugh scoring system, which was designed to predict mortality in cirrhosis patients [10]. The Child–Pugh score is determined based on multiple clinical and laboratory criteria including encephalopathy, ascites, bilirubin, albumin, and prothrombin time/internationalized normalized ratio (INR) where each criterion is associated with a pre-specified number of points based on increasing severity [10]. Patients with 5–6 points are classified as having Child–Pugh A (CPA) cirrhosis (i.e., good hepatic function); patients with 7–9 points are classified as having Child–Pugh B (CPB) cirrhosis (i.e., moderately impaired hepatic function); and patients with 10–15 points are classified as having Child–Pugh C (CPC) cirrhosis (i.e., advanced hepatic dysfunction) [10].

Notably, all studies evaluating investigational drugs as first-line therapies in advanced HCC limited their enrollment to patients with CPA cirrhosis. Furthermore, in the second-line setting (after prior treatment with sorafenib), data are also primarily limited to CPA patients, since patients with CPB and CPC cirrhosis are typically excluded from clinical studies due to their poor prognosis and low expected response rate [11]. Thus, within the expanding landscape of systemic therapies for patients with advanced HCC, management of HCC with more advanced stages of cirrhosis clearly constitutes an unmet clinical need.

## Adenosine receptors in normal physiology and various pathologies

Adenosine is a ubiquitous nucleoside present in most cell types. It is released from metabolically active or stressed cells and subsequently acts as a regulatory molecule through binding to cell surface-specific adenosine receptors (AR) termed A1AR, A2AAR, A2BAR, and A3AR [12, 13]. As nearly all cells express specific adenosine receptors, adenosine serves as an important physiological regulator with cardio-protective, neuro-protective, chemo-protective, and immunomodulator functional activities [14–19].

The cDNA encoding A3AR was cloned from a human heart library approximately 30 years ago [20]. Northern blot analysis of various human tissues showed that the human A3AR gene is expressed primarily in the lung, liver, kidney, and heart, whereas A3AR expression in the brain and skeletal muscles is very limited [20, 21]. The A3AR is over-expressed in tumor and inflammatory cells [22–27]. For example, a study examining A3AR expression (using reverse transcription polymerase chain reaction [RT-PCR] and Western blot analyses) in tumor tissues, adjacent normal tissues, and in regional lymph node metastases from patients with colon or breast carcinoma demonstrated that colon and breast carcinoma tissues had higher A3AR expression and higher A3AR protein levels compared to the adjacent healthy tissues [24]. Furthermore, lymph node metastases had even higher A3AR expression compared to the primary tumor tissue [24]. Notably, the elevated expression of A3AR is also reflected in peripheral blood cells from cancer patients (threefold higher compared with healthy subjects), as was first demonstrated nearly 20 years ago in patients with colon carcinomas [23].

Selective agonists are now available for all 4 adenosine receptor subtypes [28, 29]. Over a dozen of these selective agonists are now in clinical trials for various indications, although, thus far, none has received regulatory approval except for the endogenous AR agonist, adenosine (which is indicated not in oncology, but rather as an adjunct to thallium-201 myocardial perfusion scintigraphy in patients unable to exercise adequately [30]). A1AR agonists are in clinical trials for the treatment of cardiac arrhythmias and neuropathic pain; A2aAR agonists are in clinical trials for myocardial perfusion imaging and as anti-inflammatory agents; A2bAR agonists are in preclinical evaluation for the potential treatment of cardiac ischemia; and A3AR agonists are in clinical trials for the treatment of psoriasis, NAFLD, with or without nonalcoholic steatohepatitis (NASH), and HCC [31].

All ARs operate through distinct biochemical signaling mechanisms. The A1AR and A3AR subtypes control their cellular responses via pertussis toxin-sensitive G proteins of the Gi and Go family. A3AR triggers Gi-proteins and induces a signaling cascade that increases intracellular calcium concentrations thereby activating phospholipase C and D, inhibiting the activity of adenylyl cyclase and the production of cAMP, leading to related cellular responses such as cell proliferation or tumor cell apoptosis [24, 25, 32–38].

Indeed, molecular targeting of the A3AR by synthetic agonists results in a differential effect on tumor versus normal cells, due to the overexpression of A3AR in the former. In vitro, synthetic A3AR agonists were shown to inhibit the growth of various tumor cell types. For example, the synthetic A3AR agonist CF101 inhibited HCT-116 human colon carcinoma cells in vitro at the low nanomolar range, an effect that was reversed by a selective A3AR antagonist (MRS1523) [39]. Tumor growth inhibitory effects were also observed in vivo in experimental animal models of melanoma, prostate cancer, colon carcinoma, breast cancer, and HCC (summarized by Fishman et al. [40]). For example, CF101 inhibited the development of primary tumors in a xenograft model of HCT-116 human colon cancer and a syngeneic model of murine CT-26 colon carcinoma cells [39]. Furthermore, CF101 also significantly suppressed the development of liver metastases in the syngeneic mice where the spleen was inoculated with CT-26 cells [39]. Another example is the inhibition of the development of B16-F10 melanoma in mice (a flank model) by CF101, an effect that was reversed by the selective A3AR antagonist, MRS1523 [41].

## A3AR: a valid therapeutic target in liver diseases

The A3AR is over-expressed in human HCC tissues and peripheral blood mononuclear cells (PBMCs) derived from HCC patients. This overexpression was demonstrated in a study involving 21 patients with HCC (disease duration, 3–6 years). The overexpression in these patients was observed in the PBMCs, as well as in the tumor tissues but not in the adjacent healthy tissues [42]. The high expression level of A3AR was directly correlated to high expression levels of the transcription factor NF-κB, known to be present in the A3AR gene promoter [42]. The findings from HCC patients are consistent with preclinical pharmacology studies in N1S1 HCC tumor-bearing rats, in which overexpression of A3AR in the tumor tissue was reflected in the PBMCs of the animals [42].

## Namodenoson: a highly selective A3AR agonist

**Fig. 1:**
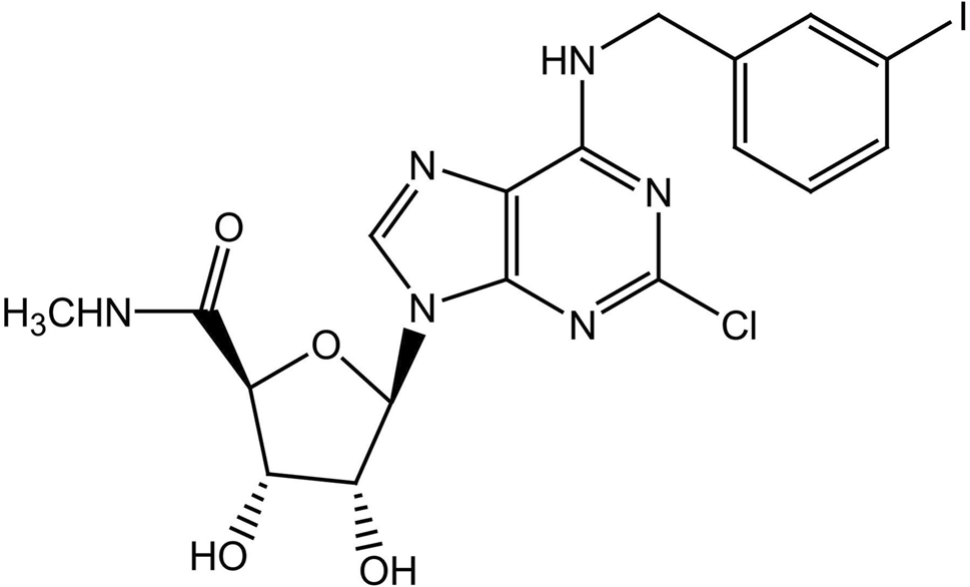
Namodenoson chemical structure

A series of highly selective A3AR agonists have been synthesized and their interactions with A3AR have been characterized using site-directed mutagenesis and molecular modeling [40]. Typical A3AR agonists are adenosine derivatives that contain 5′-uronamide and N^6^-benzyl modifications leading to nanomolar receptor affinity [40]. Namodenoson (CF102, CL-IB-MECA, Can-Fite BioPharma, Petah Tikva, Israel) is a 2-chloro analog of the protoypical agonist CF101. Its molecular formula is C_18_H_18_CIN_6_O_4_, and its molecular weight is 544.73 Da. The molecular structure of namodenoson is depicted in Fig. 1.

Namodenoson activity is specific and selective to A3AR. Its IC_50_ value for A3AR is 3 orders of magnitude lower compared to that for the other adenosine receptors (Table 1).

**Table 1:** Binding of Namodenoson to Human Receptors, as determined with radioligand binding assays

Receptor	IC_50_ (nM)	K_i_ (nM)^a^
A_1_	5,390	3,140
A_2a_	2,090	1,170
A_2b_	No activity	No activity
A_3_	0.717	0.661

^a^544.73 ng/mL = 1 μM

### Namodenoson: preclinical pharmacology studies

**Fig. 2:**
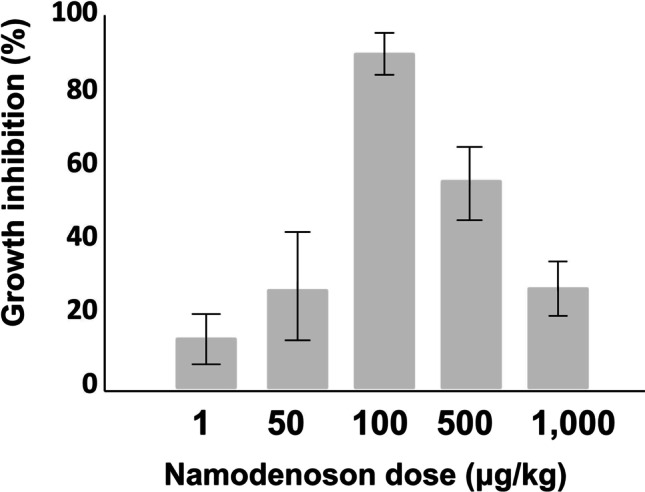
Effect of namodenoson on the growth of HCC tumors in a rat orthotopic model [42]

A3AR agonists were shown to inhibit tumor cell growth in vitro in the N1S1 and Hep-3b HCC cell lines [42, 43]. In HCC xenograft and orthotopic models, namodenoson given orally to tumor-bearing animals inhibited tumor growth remarkably [42]. Interestingly, in the N1S1 orthotopic model where 1, 50, 100, 500, and 1000 μg/kg were introduced thrice daily to the tumor-bearing animals, a bell-shaped effect was observed, with a maximal effect at the 100 μg/kg dose (Fig. 2) [42].

These preclinical data suggest that A3AR agonists cause a clear linear dose–response curve at low doses. A downturn of the dose–response curve occurs when ligand dose increases, resulting in lower efficacy due to decreased receptor response to the agonist.

The bell-shaped dose–response curve is well established for drugs that target other G protein-coupled receptors. For example, buprenorphine, a partial agonist at the ORL1/nociceptin G protein-coupled μ-opioid receptor, which is used to treat opiod dependence, as an analgesic, and as an antiemetic following chemotherapy, also exhibits a bell-shaped dose–response curve [44–46].

Also, upon treatment of the N1S1-bearing animals with namodenoson, the A3AR expression level was downregulated in the excised tumor lesions and the PBMCs. Receptor downregulation represents a response to the agonist due to receptor internalization and degradation in the lysosome [42]. It is well established that Gi protein receptors are internalized to early endosomes upon agonist binding. Studies with B-16 melanoma cells showed that A3AR is then re-synthesized in the cells and externalized to the cell membrane, ready for interaction with the agonist [41, 42].

Namodenoson demonstrated a favorable safety profile in nonclinical toxicology testing and is an orally bioavailable small molecule (Can-Fite BioPharma Ltd., data on file), characteristics that make it an attractive candidate as a human therapeutic agent.

### Namodenoson: mechanism of action in HCC

**Fig. 3:**
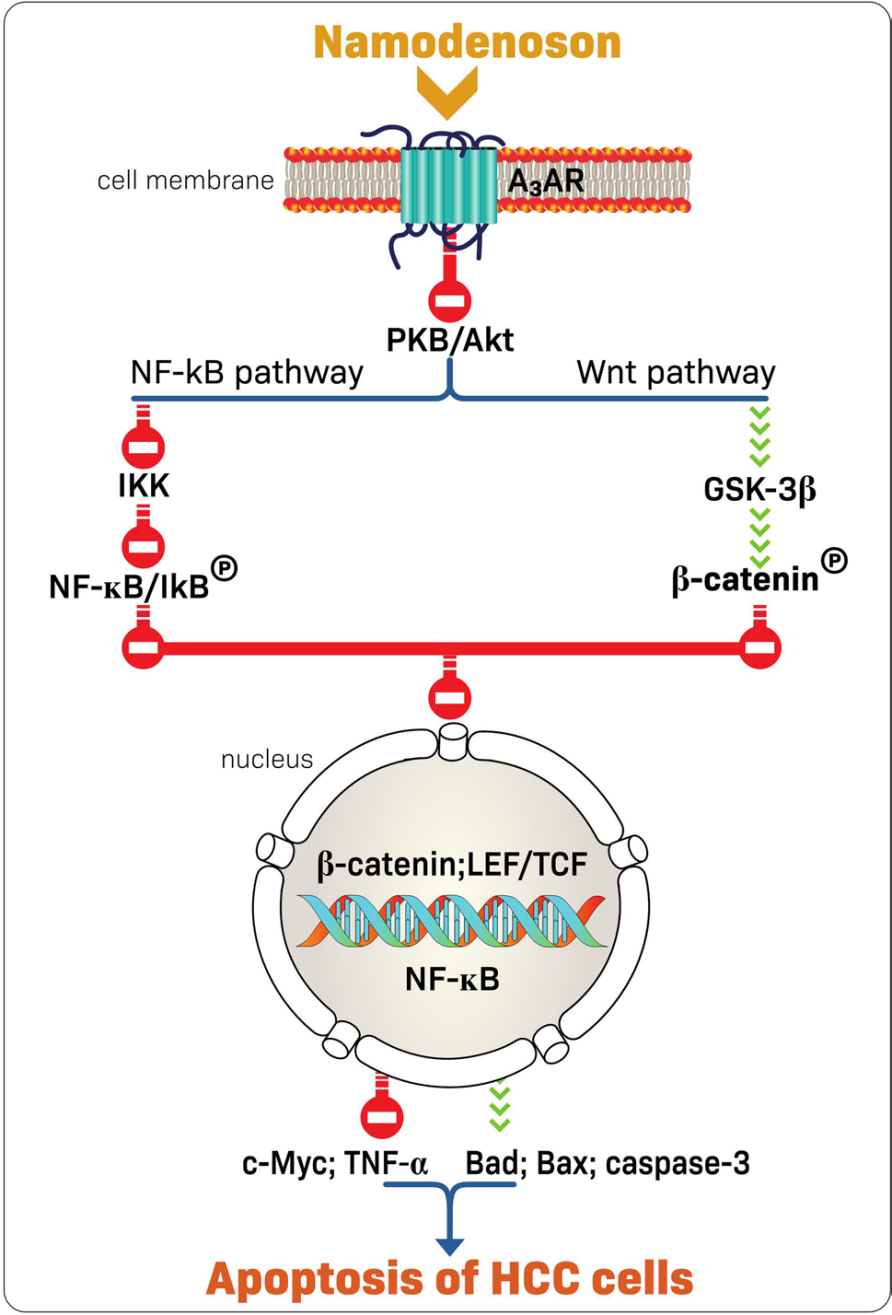
Mechanism of action of namodenoson

The molecular mechanism underlying the tumor growth inhibition activity of namodenoson involves de-regulation of the NF-κB and the Wnt signaling pathways (Fig. 3) [42]. This was demonstrated using Western blot analyses on tumor protein extracts derived from N1S1 tumor-bearing rats that were treated with namodenoson or vehicle [42]. Using the same approach, it was shown that pro-apoptotic proteins such as Bad, Bax, and caspase-3 are upregulated in the namodenoson-treated N1S1 tumor-bearing rats. In addition, the TUNEL assay performed on tumor sections derived from namodenoson- and vehicle-treated N1S1 tumor-bearing rats demonstrated abundant apoptotic cells in the namodenoson-treated but only a few apoptotic cells in the vehicle-treated animals [42]. Notably, this main mechanism of action of namodenoson is potentiated by another mechanism involving the stimulation of natural killer cells [47]. This was demonstrated in another preclinical study involving mice inoculated with B16-F10 melanoma cells, which showed growth inhibition of the melanoma in namodenoson-treated mice. The study also showed that this growth inhibition was correlated with higher serum levels of interleukin 12 and the potentiation of natural killer cells [47]. Furthermore, growth inhibition of lung metastasis loci was observed in the melanoma-bearing mice upon engraftment with splenocytes derived from namodenoson-treated mice, further supporting the role of natural killer cells in the namodenoson anticancer activity [47].

Interestingly, in contrast to the inhibitory effect of namodenoson on cancer cells, the effect of namodenoson on myeloid cells seems to be myelostimulatory. Mechanistic studies involving the A3AR agonist CF101 showed that oral administration of this agent led to an elevation of serum granulocyte colony-stimulating factor (G-CSF), as well as an increase in absolute neutrophil counts (ANC) and bone marrow colony-forming cells [48]. The molecular mechanisms associated with these effects involve the upregulation of NF-κB and the upstream kinases phosphoinositide 3-kinase (PI3K), PKB/Akt, and IKK [48].

### Protective effects of namodenoson on the liver: preclinical evidence

Namodenoson acts as a hepatoprotective agent against liver injury in models of acute hepatitis, hepatic ischemia, steatohepatitis, and partial hepatectomy. Studies in mice with hepatic inflammation induced by concanavalin A demonstrated that namodenoson reduced serum levels of the liver enzymes alanine transaminase (ALT) and aspartate aminotransferase (AST) compared to the vehicle-treated group [43].

Namodenoson was also shown to protect the liver from ischemia/reperfusion (IR) injury in a preclinical IR model in Wistar rats. Namodenoson prevented the increase in serum liver enzymes (ALT, AST) and protected the liver from apoptosis [49]. In addition, namodenoson was demonstrated to accelerate liver regeneration upon partial hepatectomy. In rats that underwent partial hepatectomy (70%) under ischemia and then received namodenoson, histopathology of the livers revealed induced mitosis of hepatocytes, increased mitotic index, and increased liver mass [49]. In addition, namodenoson was shown to have anti‑inflammatory and anti‑steatotic effects in murine models of NASH including the STAM model and the carbon tetrachloride model. In the former, namodenoson significantly decreased the NAFLD activity score, and in the latter, it reversed ALT levels to normal and significantly improved liver inflammation and fibrosis [50].

### Clinical evaluation of namodenoson in advanced HCC

#### Phase I study

In the phase I double-blind, randomized study, namodenoson was evaluated in 25 healthy male, non-smoker subjects enrolled in 5 cohorts of 5 subjects each at a 4:1 ratio (4 randomized to namodenoson and 1 to matching placebo). A period of approximately 2 weeks separated the dosing of each cohort. In this healthy volunteer trial, single doses of oral namodenoson as high as 40 mg were not associated with intolerability, clinically important adverse events (AEs), or changes in electrocardiograms or laboratory assessments (Can-Fite BioPharma Ltd., data on file).

#### Phase I/II study

In phase I/II, open-label, dose-escalation study (clinicaltrials.gov identifier: NCT00790218), namodenoson was evaluated in patients with advanced HCC. The study (CF102-102HCC) was designed to evaluate the safety, long-term tolerability, maximum tolerated dose, pharmacokinetics, pharmacodynamic effects, and preliminary clinical activity of namodenoson in this population [51]. A total of 18 patients received BID oral doses of namodenoson in consecutive, 28-day cycles. The starting dose was 1 mg BID, with subsequent escalations by cohort to 5 mg BID and 25 mg BID (each dose level included 6 patients). Of the 18 patients, 5 had CPB cirrhosis at baseline, of whom 3 received the 25 mg BID dose. Efficacy was evaluated using Response Evaluation Criteria in Solid Tumors (RECIST) v1.0. There were no objective complete responses (CR) or partial responses (PR) assessed by RECIST criteria. Four patients (22%) had a best overall response of stable disease (SD) that lasted at least 4 months. One of the patients presented with skin nodules that were HCC metastases (as proven by biopsy) and achieved a complete metastasis regression during 3 months of namodenoson treatment [51]. Median overall survival (OS) was 7.8 months for all patients, and 7.0 months for patients who progressed on sorafenib treatment. CPB patients had a median OS of 8.1 months [51].

#### Phase II study

In phase II blinded, randomized, placebo-controlled study that was designed to further explore the efficacy signal seen in the subgroups analysis of the phase I/II study, the namodenoson dose evaluated was oral 25 mg BID. The study population consisted of 78 patients with advanced HCC but was specifically limited to patients with CPB cirrhosis who did not tolerate sorafenib or whose disease progressed despite first-line therapy with sorafenib (clinicaltrials.gov identifier: NCT02128958). Patients were randomized in a 2:1 ratio to receive namodenoson 25 mg BID (*n* = 50) or placebo (*n* = 28) [52].

The safety and tolerability experience in this study was consistent with the favorable profile seen in earlier trials. No treatment-related deaths were reported. Also, no patients withdrew from the study and no dose reductions were attributable to namodenoson. Importantly, no hepatotoxicity was reported and liver function tests demonstrated no adverse namodenoson-related effect. Mean serum albumin levels and albumin-bilirubin (ALBI) scores also did not change significantly in both arms throughout the study. Only one grade 3 treatment-related AE was reported (hyponatremia) [52].

**Fig. 4:**
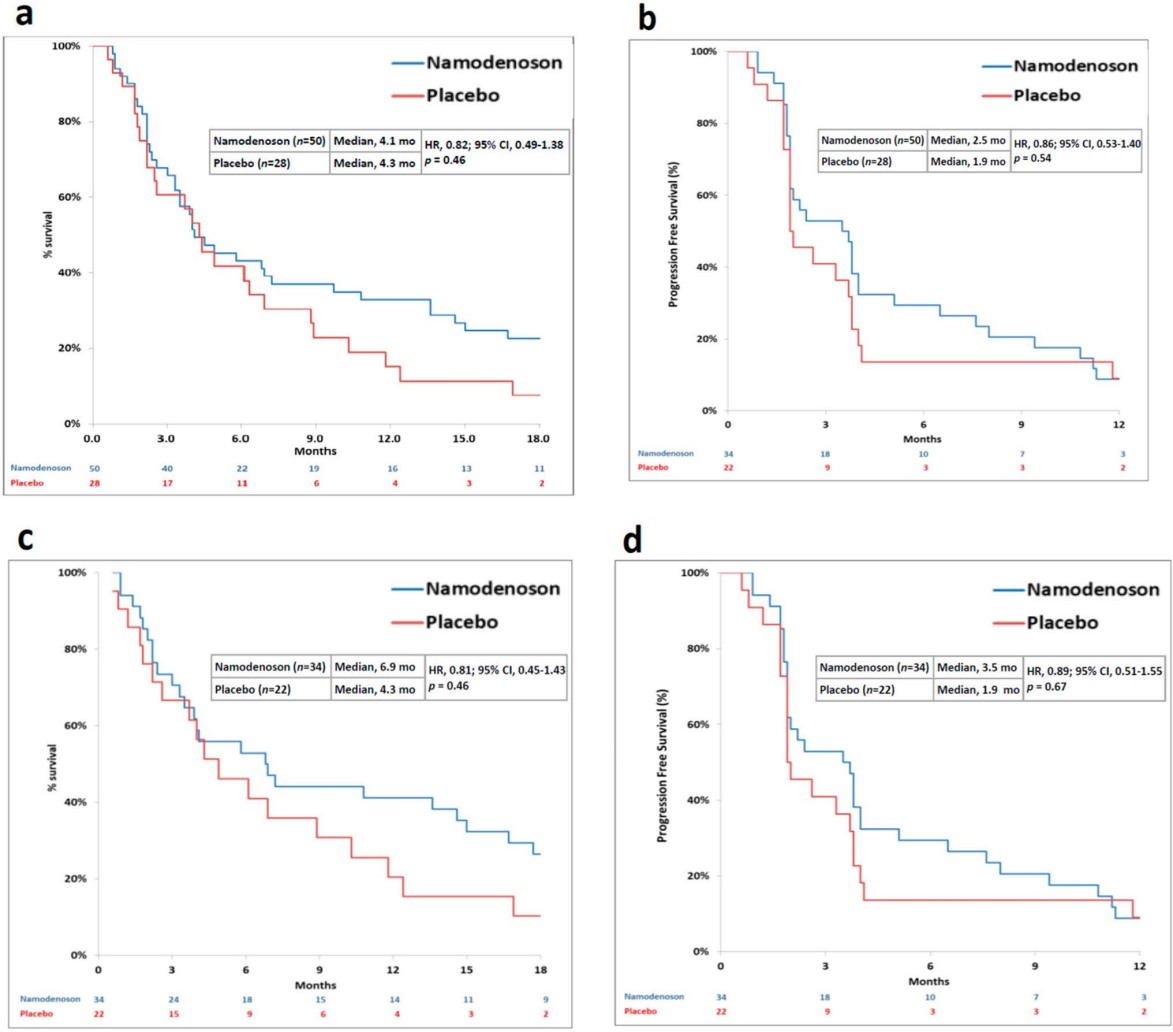
Kaplan–Meier curves by treatment group for all patients and Child–Pugh Score 7 (CPB7) patients in the phase II Trial. **a** overall survival (OS) in all patients; **b** progression-free survival (PFS) in all patients; **c** OS in CPB7 patients; **d** PFS in CPB7 patients. Reprinted from Stemmer et al. [52]

The primary trial efficacy endpoint of OS superiority over placebo was not met; median OS was 4.1 and 4.3 months for namodenoson and placebo, respectively (hazard ratio [HR], 0.82; 95% confidence interval [CI] 0.49‒1.38; *p* = 0.46) (Fig. 4a). Similarly, no superiority was observed for progression-free survival (PFS) (Fig. 4b). In this CBP study population, patients with a Child–Pugh score of 7 (the least severe form of hepatic dysfunction within the CPB category) were the largest group (34 patients in the namodenoson arm and 22 patients in the placebo arm). Preplanned analysis in which patients were evaluated by Child–Pugh subgroups demonstrated nonsignificant differences in OS and PFS for patients with a Child–Pugh score of 7. In this subcategory, the median OS was 6.9 months in the namodenoson group vs 4.3 months in the placebo group (HR, 0.81; 95% CI 0.45‒1.43; *p* = 0.46). The median PFS was 3.5 months vs 1.9 months (HR, 0.89; 95% CI 0.51‒1.55; *p* = 0.67) (Fig. 4c, d). In patients with a Child–Pugh score of 8 (13 patients; 7 namodenoson-treated and 6 placebo-treated), OS and PFS were similar between the namodenoson and placebo arms and were overall shorter than those reported for the subgroup of patients with a Child–Pugh score of 7 (OS: 3.3 vs 3.4 months, for the namodenoson and placebo arms respectively; HR, 0.88; 95% CI 0.28‒2.77, *p* = 0.83. PFS: 2.1 vs 1.9 months, respectively; HR, 0.71; 95% CI 0.23‒2.17, *p* = 0.53). The median OS and PFS values for the 9 patients with a Child–Pugh Score of 9, who were all in the namodenoson arm, were 3.5 and 2.2 months, respectively, similar to those in patients with a Child–Pugh score of 8 [52]. Exploratory analysis comparing OS by treatment arm and stratification by gender, alfa-fetoprotein levels, Eastern Cooperative Oncology Group (ECOG) Performance Status (PS), HPB, and HPC status, locoregional therapy, extrahepatic spread status, and portal vein thrombosis status found no statistically significant differences between the study arms in any of the subgroups, which could be attributed, in part, to the relatively small sample size in some of the groups [52].

**Fig. 5:**
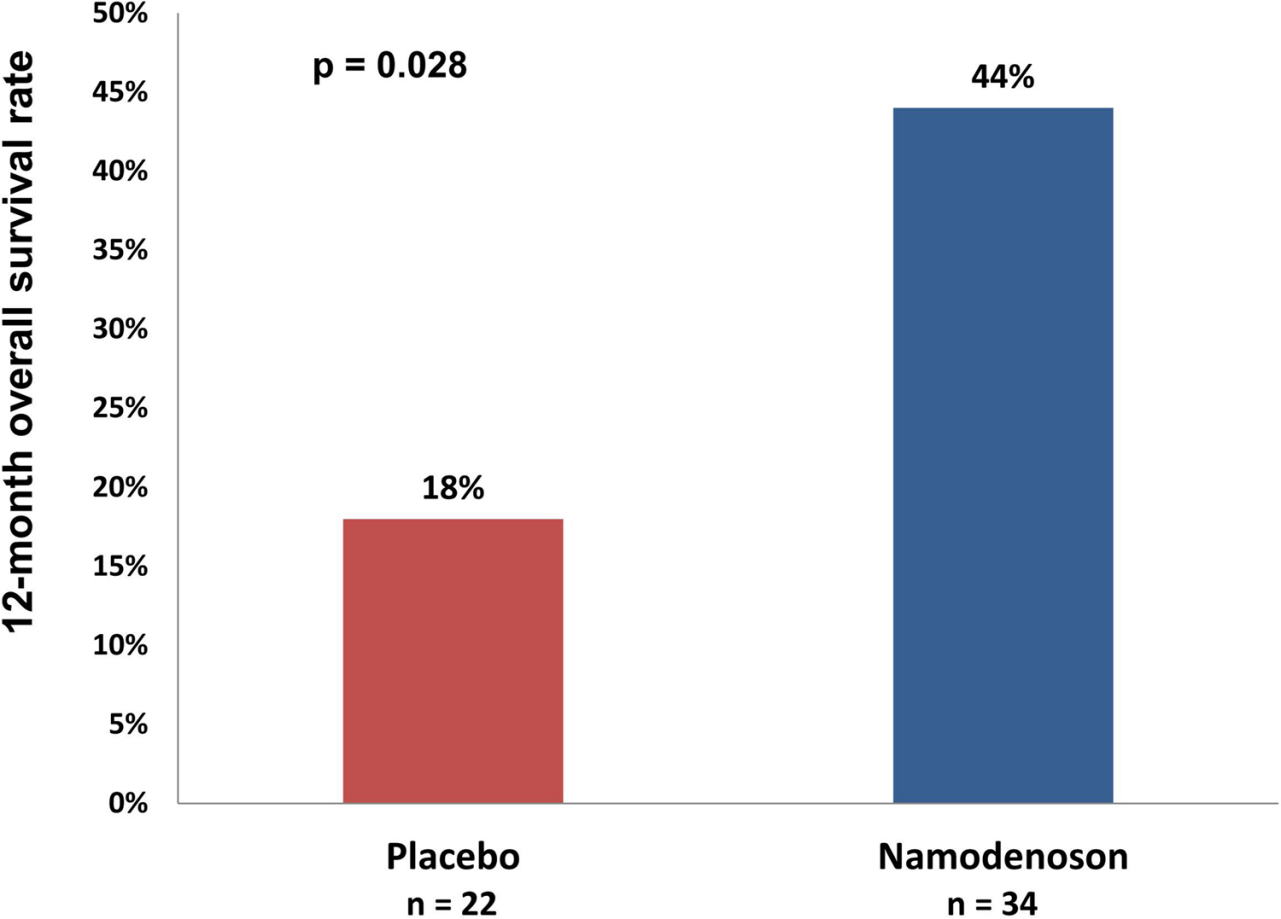
Comparison of 12-month overall survival (OS) rate in patients with Child–Pugh score 7 (namodenoson 25 mg BID vs placebo) in the phase II study [52]

The difference in the 12-month OS rate was statistically significant (44% and 18% in the namodenoson and placebo arms, respectively; *p* = 0.028) (Fig. 5). Analysis of the response in all patients for whom at least 1 post-baseline assessment was available (55 patients; 34 namodenoson-treated and 21 placebo-treated) revealed that no patient experienced CR and that PR was achieved by 3 patients (9%) in the namodenson group vs none in the placebo group (Table 2) [52]. In the 3 patients who experienced PR, the duration of response was 2, 6, and 26 months [52].

**Table 2:** Best Observed responses (RECIST 1.1) by treatment arm in the phase II trial [52]

Response, *n* (%)	Namodenoson *n* = 34	Placebo *n* = 21
CR	0 (0.0%)	0 (0.0%)
PR	3 (8.8%)	0 (0.0%)
SD	17 (50.0%)	10 (47.6%)
PD	14 (41.2%)	11 (52.4%)

Abbreviations: *CR*, complete response; *PD*, progressive disease; *PR*, partial response; *RECIST*, response evaluation criteria in solid tumors; *SD*, stable disease

### Clinical evaluation in other liver diseases

As the preclinical studies suggested that namodenoson is characterized by anti-tumor *and* hepatoprotective effects [50], namodenoson has also been clinically evaluated as a treatment for NAFLD with or without NASH. A phase II double-blind placebo-controlled dose-finding study investigated namodenoson in patients with NAFLD and serum ALT levels ≥ 60 U/L (clinicaltrials.gov identifier: NCT02927314) [53]. Sixty patients were randomized (1:1:1) to receive oral namodenoson 25 mg BID, 12.5 mg BID, or placebo BID for 12 weeks and were followed up until week 16. The safety profile for namodenoson in this study was favorable and consistent with other namodenoson studies. Dose dependency was observed and namodenoson 25 mg BID was more efficacious for treating NAFLD than 12.5 mg BID. Namodenoson was found to favorably affect liver function and liver morphology. Normalization of ALT levels at week 16 was reported in 37% of patients in the 25 mg BID group vs 10% in the placebo group (*p* = 0.038). Change from baseline (CFB) in AST levels was also statistically significant in the 25 mg BID group compared to placebo (week 12 mean CFB:-8.4 vs − 0.8 U/L; *p* = 0.03). Other endpoints suggesting liver-protective and anti-fibrotic effects for namodenoson included reduced liver fat volume (week 12 mean CFB: − 159 vs − 74 cm^3^ for 25 mg BID and placebo, respectively; *p* = 0.03) and decreased Fib4-scores (week 12 mean CFB: − 0.28 vs − 0.03 for 25 mg and placebo, respectively; *p* = 0.011) [53]. Phase IIb evaluating namodenoson 25 mg BID in this indication is currently underway (clinicaltrials.gov identifier: NCT04697810).

## Current regulatory status and future clinical trials

The US FDA granted an Orphan Drug and Fast Track Status to namodenoson for the treatment of HCC. The European Medicines Agency (EMA) also granted an orphan drug status to namodenoson.

A pivotal phase III clinical study has been designed and is underway (clinicaltrials.gov identifier: NCT05201404) [54]. It is a multicenter, randomized, double-blind, and placebo-controlled study of the efficacy and safety of namodenoson (25 mg BID) for treating advanced HCC in patients (≥ 18 years) with CPB cirrhosis and Child–Pugh score 7 whose disease has progressed on at least one first-line therapy. The study is designed to enroll a total of 471 patients, randomized in a 2:1 ratio (namodenoson:placebo). Treatment is administered for consecutive 28-day cycles, until disease progression or drug-related intolerability. The primary efficacy endpoint of the study is OS. Other endpoints include PFS, time-to-progression (TTP), and response rates. The design of this phase III study has been agreed upon with the FDA and EMA, and results are expected in 2025 [54].

## Conclusions

Namodenoson is a promising investigational drug with a novel mechanism of action for the treatment of liver diseases, most notably HCC patients with CPB cirrhosis and a Child–Pugh score of 7. Phase III trial in this indication is underway.

## Data Availability

Not applicable.

## References

[1] Sung H, Ferlay J, Siegel RL, Laversanne M, Soerjomataram I, Jemal A, Bray F (2021). Global cancer statistics 2020: GLOBOCAN estimates of incidence and mortality worldwide for 36 cancers in 185 countries. CA Cancer J Clin.

[2] Yang JD, Hainaut P, Gores GJ, Amadou A, Plymoth A, Roberts LR (2019). A global view of hepatocellular carcinoma: trends, risk, prevention and management. Nat Rev Gastroenterol Hepatol.

[3] Tabrizian P, Jibara G, Shrager B, Schwartz M, Roayaie S (2015). Recurrence of hepatocellular cancer after resection: patterns, treatments, and prognosis. Ann Surg.

[4] Lee MW, Raman SS, Asvadi NH, Siripongsakun S, Hicks RM, Chen J, Worakitsitisatorn A, McWilliams J, Tong MJ, Finn RS, Agopian VG, Busuttil RW, Lu DSK (2017). Radiofrequency ablation of hepatocellular carcinoma as bridge therapy to liver transplantation: a 10-year intention-to-treat analysis. Hepatology.

[5] Lencioni R, de Baere T, Soulen MC, Rilling WS, Geschwind JF (2016). Lipiodol transarterial chemoembolization for hepatocellular carcinoma: a systematic review of efficacy and safety data. Hepatology.

[6] Lobo L, Yakoub D, Picado O, Ripat C, Pendola F, Sharma R, ElTawil R, Kwon D, Venkat S, Portelance L, Yechieli R (2016). Unresectable hepatocellular carcinoma: radioembolization versus chemoembolization: a systematic review and meta-analysis. Cardiovasc Intervent Radiol.

[7] Nexavar^®^ (sorafenib) [package insert]. Whippany, NJ: Bayer Healthcare Pharmaceuticals Inc.; 2018

[8] Lenvima^®^ (lenvatinib) [Package Insert]. WoodCliff Lake, NJ: Eisai Inc.; 2021

[9] Rimassa L, Worns MA (2020). Navigating the new landscape of second-line treatment in advanced hepatocellular carcinoma. Liver Int.

[10] Jamil Z, Perveen S, Khalid S, Aljuaid M, Shahzad M, Ahmad B, Waheed Y (2022) Child-Pugh Score, MELD Score and Glasgow Blatchford Score to Predict the In-Hospital Outcome of Portal Hypertensive Patients Presenting with Upper Gastrointestinal Bleeding: An Experience from Tertiary Healthcare System. J Clin Med 11(22):6654–6665. 10.3390/jcm11226654 10.3390/jcm11226654 PMC9693334 36431131

[11] Llovet JM, Di Bisceglie AM, Bruix J, Kramer BS, Lencioni R, Zhu AX, Sherman M, Schwartz M, Lotze M, Talwalkar J, Gores GJ, Panel of Experts in HCCDCT (2008). Design and endpoints of clinical trials in hepatocellular carcinoma. J Natl Cancer Inst.

[12] Olah ME, Stiles GL (1995). Adenosine receptor subtypes: characterization and therapeutic regulation. Annu Rev Pharmacol Toxicol.

[13] Poulsen SA, Quinn RJ (1998). Adenosine receptors: new opportunities for future drugs. Bioorg Med Chem.

[14] Cohen MV, Downey JM (2008). Adenosine: trigger and mediator of cardioprotection. Basic Res Cardiol.

[15] Wardas J (2002). Neuroprotective role of adenosine in the CNS. Pol J Pharmacol.

[16] Fishman P, Bar-Yehuda S, Farbstein T, Barer F, Ohana G (2000). Adenosine acts as a chemoprotective agent by stimulating G-CSF production: a role for A1 and A3 adenosine receptors. J Cell Physiol.

[17] Fishman P, Bar-Yehuda S, Barer F, Madi L, Multani AS, Pathak S (2001). The A3 adenosine receptor as a new target for cancer therapy and chemoprotection. Exp Cell Res.

[18] Bar-Yehuda S, Madi L, Barak D, Mittelman M, Ardon E, Ochaion A, Cohn S, Fishman P (2002). Agonists to the A3 adenosine receptor induce G-CSF production via NF-kappaB activation: a new class of myeloprotective agents. Exp Hematol.

[19] Bar-Yehuda S, Madi L, Silberman D, Gery S, Shkapenuk M, Fishman P (2005). CF101, an agonist to the A3 adenosine receptor, enhances the chemotherapeutic effect of 5-fluorouracil in a colon carcinoma murine model. Neoplasia.

[20] Sajjadi FG, Firestein GS (1993). cDNA cloning and sequence analysis of the human A3 adenosine receptor. Biochim Biophys Acta.

[21] Zhou QY, Li C, Olah ME, Johnson RA, Stiles GL, Civelli O (1992). Molecular cloning and characterization of an adenosine receptor: the A3 adenosine receptor. Proc Natl Acad Sci U S A.

[22] Fishman P, Madi L, Bar-Yehuda S, Barer F, Del Valle L, Khalili K (2002). Evidence for involvement of Wnt signaling pathway in IB-MECA mediated suppression of melanoma cells. Oncogene.

[23] Gessi S, Cattabriga E, Avitabile A, Gafa R, Lanza G, Cavazzini L, Bianchi N, Gambari R, Feo C, Liboni A, Gullini S, Leung E, Mac-Lennan S, Borea PA (2004). Elevated expression of A3 adenosine receptors in human colorectal cancer is reflected in peripheral blood cells. Clin Cancer Res.

[24] Madi L, Ochaion A, Rath-Wolfson L, Bar-Yehuda S, Erlanger A, Ohana G, Harish A, Merimski O, Barer F, Fishman P (2004). The A3 adenosine receptor is highly expressed in tumor versus normal cells: potential target for tumor growth inhibition. Clin Cancer Res.

[25] Bar-Yehuda S, Silverman MH, Kerns WD, Ochaion A, Cohen S, Fishman P (2007). The anti-inflammatory effect of A3 adenosine receptor agonists: a novel targeted therapy for rheumatoid arthritis. Expert Opin Investig Drugs.

[26] Fishman P, Jacobson KA, Ochaion A, Cohen S, Bar-Yehuda S (2007). The anti-cancer effect of A3 adenosine receptor agonists: a novel, targeted therapy. Immun Endoc Metab Agents Med Chem.

[27] Madi L, Cohen S, Ochayin A, Bar-Yehuda S, Barer F, Fishman P (2007). Overexpression of A3 adenosine receptor in peripheral blood mononuclear cells in rheumatoid arthritis: involvement of nuclear factor-kappaB in mediating receptor level. J Rheumatol.

[28] Olah ME, Stiles GL (2000). The role of receptor structure in determining adenosine receptor activity. Pharmacol Ther.

[29] Gao ZG, Jacobson KA (2007). Emerging adenosine receptor agonists. Expert Opin Emerg Drugs.

[30] Adenoscan (adenosine injection) [package insert]. Lake Forest, IL: Hospira, Inc.; 2020

[31] Effendi WI, Nagano T, Kobayashi K, Nishimura Y (2020). Focusing on adenosine receptors as a potential targeted therapy in human diseases. Cells.

[32] Yamano K, Inoue M, Masaki S, Saki M, Ichimura M, Satoh M (2005). Human adenosine A(3) receptor leads to intracellular Ca(2+) mobilization but is insufficient to activate the signaling pathway via phosphoinositide 3-kinase gamma in mice. Biochem Pharmacol.

[33] Appel E, Kazimirsky G, Ashkenazi E, Kim SG, Jacobson KA, Brodie C (2001). Roles of BCL-2 and caspase 3 in the adenosine A3 receptor-induced apoptosis. J Mol Neurosci.

[34] Duann P, Ho TY, Desai BD, Kapoian T, Cowen DS, Lianos EA (2005). Mesangial cell apoptosis induced by stimulation of the adenosine A3 receptor: signaling and apoptotic events. J Investig Med.

[35] Lu J, Pierron A, Ravid K (2003). An adenosine analogue, IB-MECA, down-regulates estrogen receptor alpha and suppresses human breast cancer cell proliferation. Cancer Res.

[36] Merighi S, Mirandola P, Milani D, Varani K, Gessi S, Klotz KN, Leung E, Baraldi PG, Borea PA (2002). Adenosine receptors as mediators of both cell proliferation and cell death of cultured human melanoma cells. J Invest Dermatol.

[37] Zhao Z, Kapoian T, Shepard M, Lianos EA (2002). Adenosine-induced apoptosis in glomerular mesangial cells. Kidney Int.

[38] Barcz E, Sommer E, Janik P, Marianowski L, Skopinska-Rozewska E (2000). Adenosine receptor antagonism causes inhibition of angiogenic activity of human ovarian cancer cells. Oncol Rep.

[39] Ohana G, Bar-Yehuda S, Arich A, Madi L, Dreznick Z, Rath-Wolfson L, Silberman D, Slosman G, Fishman P (2003). Inhibition of primary colon carcinoma growth and liver metastasis by the A3 adenosine receptor agonist CF101. Br J Cancer.

[40] Fishman P, Bar-Yehuda S, Liang BT, Jacobson KA (2012). Pharmacological and therapeutic effects of A3 adenosine receptor agonists. Drug Discov Today.

[41] Madi L, Bar-Yehuda S, Barer F, Ardon E, Ochaion A, Fishman P (2003). A3 adenosine receptor activation in melanoma cells: association between receptor fate and tumor growth inhibition. J Biol Chem.

[42] Bar-Yehuda S, Stemmer SM, Madi L, Castel D, Ochaion A, Cohen S, Barer F, Zabutti A, Perez-Liz G, Del Valle L, Fishman P (2008). The A3 adenosine receptor agonist CF102 induces apoptosis of hepatocellular carcinoma via de-regulation of the Wnt and NF-kappaB signal transduction pathways. Int J Oncol.

[43] Cohen S, Stemmer SM, Zozulya G, Ochaion A, Patoka R, Barer F, Bar-Yehuda S, Rath-Wolfson L, Jacobson KA, Fishman P (2011). CF102 an A3 adenosine receptor agonist mediates anti-tumor and anti-inflammatory effects in the liver. J Cell Physiol.

[44] Lutfy K, Cowan A (2004). Buprenorphine: a unique drug with complex pharmacology. Curr Neuropharmacol.

[45] Skurtveit S, Furu K, Kaasa S, Borchgrevink PC (2009). Introduction of low dose transdermal buprenorphine – did it influence use of potentially addictive drugs in chronic non-malignant pain patients?. Eur J Pain.

[46] Milne M, Crouch BI, Caravati EM (2009). Buprenorphine for opioid dependence. J Pain Palliat Care Pharmacother.

[47] Harish A, Hohana G, Fishman P, Arnon O, Bar-Yehuda S (2003). A3 adenosine receptor agonist potentiates natural killer cell activity. Int J Oncol.

[48] Merimski O, Bar-Yehuda S, Madi L, Fishman P (2003). Modulation of the A3 adenosine receptor by low agonist concentration induces antitumor and myelostimulatory effects. Drug Dev Res.

[49] Ohana G, Cohen S, Rath-Wolfson L, Fishman P (2016). A3 adenosine receptor agonist, CF102, protects against hepatic ischemia/reperfusion injury following partial hepatectomy. Mol Med Rep.

[50] Fishman P, Cohen S, Itzhak I, Amer J, Salhab A, Barer F, Safadi R (2019). The A3 adenosine receptor agonist, namodenoson, ameliorates nonalcoholic steatohepatitis in mice. Int J Mol Med.

[51] Stemmer SM, Benjaminov O, Medalia G, Ciuraru NB, Silverman MH, Bar-Yehuda S, Fishman S, Harpaz Z, Farbstein M, Cohen S, Patoka R, Singer B, Kerns WD, Fishman P (2013). CF102 for the treatment of hepatocellular carcinoma: a phase I/II, open-label, dose-escalation study. Oncologist.

[52] Stemmer SM, Manojlovic NS, Marinca MV, Petrov P, Cherciu N, Ganea D, Ciuleanu TE, Pusca IA, Beg MS, Purcell WT, Croitoru AE, Ilieva RN, Natosevic S, Nita AL, Kalev DN, Harpaz Z, Farbstein M, Silverman MH, Bristol D, Itzhak I, Fishman P (2021). Namodenoson in advanced hepatocellular carcinoma and Child-Pugh B cirrhosis: randomized placebo-controlled clinical trial. Cancers (Basel).

[53] Safadi R, Braun M, Francis A, Milgrom Y, Massarwa M, Hakimian D, Hazou W, Issachar A, Harpaz Z, Farbstein M, Itzhak I, Lev-Cohain N, Bareket-Samish A, Silverman MH, Fishman P (2021). Randomised clinical trial: a phase 2 double-blind study of namodenoson in non-alcoholic fatty liver disease and steatohepatitis. Aliment Pharmacol Ther.

[54] Can-Fite BioPharma. Namodenoson in the treatment of advanced hepatocellular carcinoma in patients with Child-Pugh class B7 cirrhosis (LIVERATION). Available from: https://clinicaltrials.gov/ct2/show/record/NCT05201404. Accessed, August 24, 2022

